# Multi-Occurrence of Twenty Mycotoxinsin Pasta and a Risk Assessment in the Moroccan Population

**DOI:** 10.3390/toxins10110432

**Published:** 2018-10-26

**Authors:** Youssef Bouafifssa, Lara Manyes, Mohamed Rahouti, Jordi Mañes, Houda Berrada, Abdellah Zinedine, Mónica Fernández-Franzón

**Affiliations:** 1 Department of Biology, Faculty of Sciences, University Mohammed V Agdal, Avenue Ibn Batouta, Rabat 10010, Morocco; youssef1238@gmail.com (Y.B.); medrahouti@gmail.com (M.R.); 2 Laboratory of Food Chemistry and Toxicology, Faculty of Pharmacy, University of València, Vicent Andrés Estelléss/n, 46100 Burjassot, Spain; lara.manyes@uv.es (L.M.); jordi.manes@uv.es (J.M.); monica.fernandez@uv.es (M.F.-F.); 3 Team of Applied Microbiology and Biotechnologies, Laboratory of Marine Biotechnologies and Environment (BIOMARE), Faculty of Sciences, Chouaïb Doukkali University, El Jadida 24000, Morocco; zinedineab@yahoo.fr

**Keywords:** mycotoxins, occurrence, QuEChERS, pasta, risk assessment

## Abstract

In the present study, the multi-occurrence of twenty (20) mycotoxins in pasta samples consumed in Morocco was assessed. For this, a modified *Quick*, *Easy*, *Cheap Effective*, *Rugged*, and *Safe* method was validated. The mycotoxins studied were identified and quantified by liquid chromatography–tandem mass spectrometry (LC–MS/MS) and gas chromatography–tandem mass spectrometry (GC-MS/MS). The validated method was applied to one hundred and six (*n* = 106) pasta samples purchased from several areas in the country. The analytical results showed that 99 out of 106 total samples (93.4%) were contaminated with at least one mycotoxin. Nine mycotoxins (Aflatoxin B1, Enniatin B, Enniatin B1, Enniatin A1, Zearalenone, Deoxynivalenol, 3-Acetyl-Deoxynivalenol, T-2, and HT-2 toxins) were present in the pasta samples. Enniatin B and Enniatin B1 were the predominant mycotoxins. The Zearalenone, Deoxynivalenol, HT-2, and T-2 toxins were present in 51.8%, 43.5%, 34.9%, and 16% of samples, respectively. Aflatoxin B1 was detected in only 2 samples. Risk exposure assessment concluded that mycotoxin levels found in pasta do not pose a significant human health risk for the Moroccan population. This is the first paper drafted on the multi-occurrence of mycotoxins in pasta from this country.

## 1. Introduction

Pasta is a cereal-based food produced and consumed all over the world. It is made from rice flour in Asia, from maize flour in South America, and from wheat in Northern, Eastern Europe, and Africa, but in general, pasta is made of durum wheat semolina. According to the International Association of Pasta, about 13.6 million tons of pasta are produced worldwide by more than 45 producer countries [1]. Pasta plays an important role in human nutrition and is present on a daily basis. It is easy to prepare and has many advantages for consumers such as having a low price, excellent nutritional value, multiple ways of preparation, and the possibility of being stored for a long time period after production [2]. Moroccan population is one of the largest consumers of cereals and cereal products. Indeed, 6 million tons of cereals are consumed each year and the average consumption of cereals is 200 kg per capita. Pasta constitutes one of the most important cereal-based products of the Moroccan economy since the production of pasta in the country reached about 85,000 tons in 2009 [3].

Pasta can be contaminated with mycotoxins that are natural food and feed contaminants, produced mainly by the molds of the genera *Aspergillus*, *Penicillium*, and *Fusarium*. Since pasta is very commonly used in the human diet, mycotoxins are of concern due to their potentially harmful effects. Mycotoxins are currently considered the most important chronic dietary risk factor, higher than synthetic contaminants, food additives, or pesticide residues [4]. Aflatoxin B1 (AFB1) and other naturally occurring aflatoxins (AFs) have been classified as group 1 human carcinogens; ochratoxin A (OTA) and fumonisins are classified in group 2B as possible human carcinogens [5]. Monitoring programs indicate that mycotoxin contamination is a worldwide problem [6] since it causes economic losses, both for the grain and for the marketing of foods and feeds, so it is a potential threat to animal and human health [7]. The quality and safety of the final product depend on the raw materials, the type of product processing, and the conditions used in pasta preparation [8,9]. Indeed, the technological process of producing pasta and pasta related products (thermal treatment and drying processes) will not eliminate all the microorganisms such as molds producing mycotoxins, or mycotoxins. In addition, during the production process, some microorganisms or mycotoxins could contaminate the pasta [10].

Several countries have set strict regulations about the maximum limits (ML) for mycotoxins in food commodities. The European Union (EU) has established the ML at 2 and 4 µg kg^−1^ for aflatoxin B1 (AFB1) and total aflatoxins (AFTs), respectively, in cereals and cereals products. The ML of *Fusarium* mycotoxins such as Deoxynivalenol (DON), Zearalenone (ZEA), and Fumonisins are set from 200 to 1750 µg kg^−1^ in cereals and cereal-based products; however, an ML of 25 µg kg^−1^ for the sum of the toxins T-2 and HT-2 is established in pasta [11,12]. Recently, new regulations have been adopted by Moroccan authorities to set the maximum permissible limits for mycotoxins in foodstuffs intended for human consumption. The ML of AFB1 and AFTs are set at 2 and 4 µg kg^−1^ in cereals and cereal-based products, respectively. The ML of OTA is established at 5 and 3 µg kg^−1^ in raw cereals and cereal-based products, respectively. The ML of DON in raw corn and baby foods are set at 1750 and 200 µg kg^−1^, respectively. Finally, The ML of ZEA in raw corn and baby foods are established at 350 and 20 µg kg^−1^, respectively [13]. The presence of mycotoxins in cereals and cereal-based products from Morocco was already reviewed [14], however, until now, no information is available on the multi-mycotoxin occurrence in pasta commercialized in the country.

The QuEChERS (acronym of Quick, Easy, Cheap, Effective, Rugged, and Safe) method was originally developed for the determination of pesticides in vegetables. However, it has been undergoing modifications since its first application in terms of reagents, proportions, and cleanup depending on the analytes and matrices until it reaches the current application for satisfactory mycotoxin extractions. The proposed procedure evaluates mycotoxins QuEChERS extraction from pasta by avoiding the cleanup step.

Recently, increased efforts have been made to perform and develop analytical methods for the detection of low levels of mycotoxins in cereal and derivatives samples, and for the simultaneous analysis of different mycotoxins by using both liquid chromatography–tandem mass spectrometry (LC–MS/MS) and gas chromatography–tandem mass spectrometry (GC-MS/MS) [15,16].

The aims of this work were the development and the validation of a QuEChERS extraction procedure for the analysis of twenty (20) mycotoxins (four Aflatoxins, four Enniatins, three Fumonisins, Beauvericin, and Zearalenone, Nivalenol, Deoxynivalenol, Fusarenon X, 15-acetyldeoxynivalenol, and 3-acetyldeoxynivalenol, T-2, and HT-2) by LC-MS/MS and GC/MS/MS. The validated method was then applied to pasta samples collected in different cities in Morocco for mycotoxin analysis and the estimation of the potential contribution to the dietary exposure of the Moroccan consumers.

## 2. Results and Discussion

### 2.1. Method Validation

The extraction procedure used in this investigation was derived from the method previously applied in our laboratory for the multi-mycotoxin presence in couscous and wheat semolina samples detected by LC-MS/MS [17] and GC-MS/MS [18], respectively. As some *Fusarium* mycotoxins (FUS X, DON, HT-2, T-2, NIV, 15-ADON and 3-ADON) provided better sensitivity by GC-MS/MS than LC-MS/MS, both techniques were used in this research. However, a new validation of this method was required due to the matrix investigated (pasta) and the greater number (twenty) of mycotoxins analyzed. Table 1 and Table 2 show the validation parameters obtained.

For the LC-MS/MS analysis, LODs for the 13 mycotoxins ranged from 0.01 to 10 µg kg^−1^. The intra-day and inter-day precision were lower than 12% and 19% respectively for all studied compounds (Table 1). Recoveries ranged from 60% for FB1 and BEA to 117% for ENA, except for FB2. Correlation coefficients (r^2^) from the matrix-matched the calibration curve is shown in Table 1. As matrix effect was observed for the studied mycotoxins, for the reliable quantization, relative matrix calibration was essential to compensate matrix effect. For GC-MS/MS analysis, limits of quantification (LOQ) were lower than 10 µg kg^−1^ for all the studied mycotoxins (Table 2). The intra-day and inter-day precision were lower than 15% and 20%, respectively, for all studied compounds. All recoveries were higher than 60%. Matrix-matched calibration curves were built by spiking blank samples with selected mycotoxins before extraction. These results were mainly in a permitted range by Commission Regulation (EC) No. 401/2006.

### 2.2. Global Occurrence of Mycotoxins in Pasta

The presence of mycotoxins in the global samples is summarized in Table 3. Ninety nine (99) out of 106 total samples (93.4%) were found contaminated with at least one mycotoxin. In total, 9 out the 20 studied mycotoxins were present in the pasta samples; the mycotoxins found were AFB1, the three enniatins (ENB, ENB1, and ENA); ZEA, DON, and 3-ADON; and, finally, the T-2 and HT-2 toxins.

**Figure 1:**
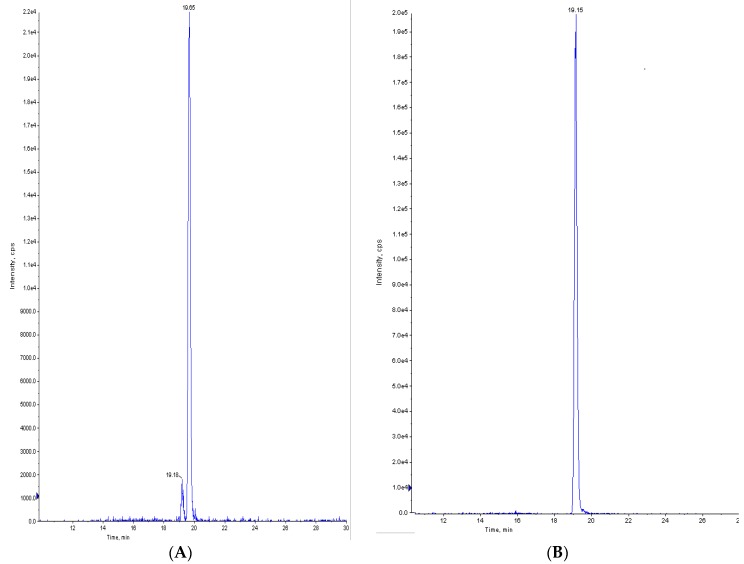
The chromatograms of two samples of pasta naturally contaminated with (**A**) enniatin B1 (1 µg kg^−1^) and (**B**) enniatin B (1.2 µg kg^−1^).

**Figure 2:**
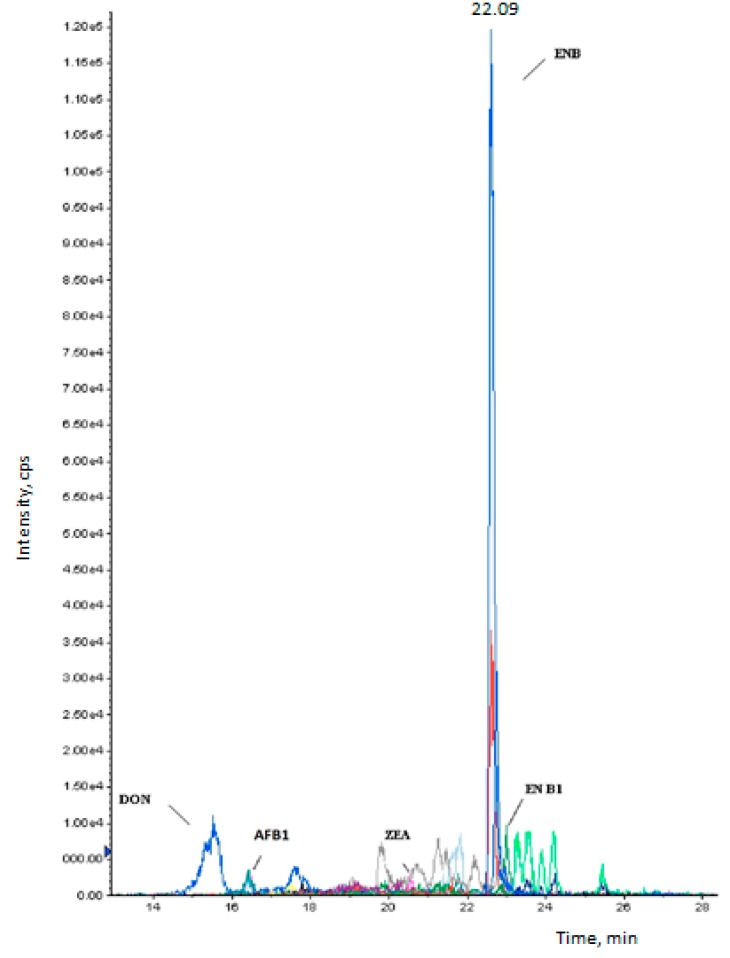
A Chromatogram of a pasta sample with the co-occurrence of five mycotoxins. DON, deoxynivalenol; AFB1, aflatoxin B1; ZEA, zearalenone; ENB1, enniatin B1; ENB, enniatin B.

The other mycotoxins studied were below the quantification limits. To our knowledge, this paper describes, for the first time, the multi-presence and the risk assessment of mycotoxins in pasta consumed in Morocco. Figure 1 shows the LC-MS/MS chromatograms for two samples of pasta naturally contaminated with ENB1 (1 µg kg^−1^) and ENB (1.2 µg kg^−1^). Figure 2 shows a chromatogram of a pasta sample with the co-occurrence of five mycotoxins (DON, AFB1, ZEA, ENB1, and ENB).

#### 2.2.1. The Occurrence of AFB1

AFB1 was present in only 2 pasta samples, one from Rabat (0.01 µg kg^−1^) and another one from Agadir (0.25 µg kg^−1^). However, AFB1 levels were below the Moroccan [13] and EU regulatory limits [11]. None of the other aflatoxins (AFB2, AFG1, and AFG2) were detected in any pasta samples. In a recent investigation from Morocco, it was reported that one corn couscous semolina was found contaminated with a high level of AFB1 (31.1 µg kg^−1^) and this sample contained a huge amount (50.7 µg kg^−1^) of the total aflatoxins [17].

#### 2.2.2. The Occurrence of ZEA

ZEA was present in 55 out of 106 samples (51.8%). All samples analyzed in this survey were below the maximum level (75 µg kg^−1^) of ZEA set by the EU regulatory limits in cereal and derivatives [12]. Nevertheless, up until now, no maximum limits of ZEA are in force in cereal products by Moroccan regulation adopted recently in 2016. Concerning the frequencies of contamination of pasta samples by origin, it has been observed that high contamination frequencies were found in samples from Agadir (81.8%), Tanger (80%), and Casablanca (76.5%). ZEA is a mycotoxin produced by the *Fusarium* species. Fungi of the genus *Fusarium* infect cereals before their harvest in the field. For comparison, a study carried out in Germany reported the ZEA occurrence in a total of 99 cereal samples (41 samples of wheat, 17 of oats, and 41 of corn) [19]. The authors reported that the incidences of ZEA were 63%, 24%, and 85%, respectively, and the mean concentrations were 15, 21, and 48 µg kg^−1^, respectively. Another study conducted in Egypt showed that ZEA was detected in some samples of wheat (40%), the levels of contamination ranged between 0.53 and 2.5 µg kg^−1^ [20].

#### 2.2.3. The Occurrence of DON and 3-ADON

DON was detected in 43 pasta samples out of 106 total samples (43.5%) with concentration levels ranging from 16 to 900 µg kg^−1^. There were 22 pasta samples (21%) contaminated with DON levels ranging from 830 to 900 µg kg^−1^ and exceeding the ML set for DON in cereal products set by the EU regulations (750 µg kg^−1^). In Morocco, there is no ML for DON in pasta; however, a schedule alert of 750 µg kg^−1^ will be applied for this mycotoxin in dry pasta by 2020 according to the Moroccan mycotoxins legislation [13].

The highest DON concentrations in the pasta were found in Agadir, Casablanca, Tanger, and Rabat samples with maximum values of 900, 830, 770, and 538 µg kg^−1^, respectively. These four cities are located on the Atlantic Ocean which is characterized by a hot and humid climate that could probably lead to mold contamination and mycotoxin production, especially in coastal areas. Concerning the toxin 3-ADON, only one pasta sample from Agadir was contaminated with a value of 3 µg kg^−1^. In a previous study, it was reported that durum wheat collected in Morocco was contaminated with the toxin DON (11% of positive samples) with concentrations that ranged from 65 to 1310 µg kg^−1^ [21]. More recently, Blesa et al. reported the presence of DON in wheat grain samples with levels that ranged between 121 and 1480 µg kg^−1^ [22].

Few reports are available worldwide on the presence of DON and derivatives in pasta samples. Indeed, De Nijs et al. reported the presence of DON in four out of 26 dry pasta samples, but none of the samples exceeded the maximum permitted level established by EU countries [8]. In Spain, the presence of DON in 479 cereal-based food products including breakfast cereals, snacks, and pasta showed that DON was the main trichothecene present in positive pasta samples [23]. A study on Italian population exposure to DON through pasta consumption showed that 78.6% of the 472 samples were contaminated with DON with a mean value of 64.8 µg kg^−1^ [24].

#### 2.2.4. The Occurrence of T-2 and HT-2 Toxins

Concerning the presence of T-2 and HT-2 in pasta, 17 (16%) and 37 (34.9%) samples were found contaminated, respectively. Contamination levels varied between 4 and 419 µg kg^−1^ for HT-2, and from 4 to 50 µg kg^−1^ for T-2. Until now, no maximum limits for T-2 or HT-2 have been adopted by Moroccan regulations. According to the analytical results, it could be observed that the incidence of the toxins T-2 and HT-2 in pasta samples is similar to the incidence of ZEA, but it is slightly higher than the incidence of DON. The high contamination of cereal grains with the T-2 and HT-2 toxins (as compared to the DON incidence) was also observed during previous studies in cereals and processed cereals available in Italy and Spain [25,26]. Recently, a low contamination level of semolina couscous samples with T-2 and HT-2 was reported [17].

#### 2.2.5. The Occurrence of Enniatins

Concerning enniatins, ENB and ENB1 were predominant in positives samples; they were present in 72 out of 106 total pasta samples (67.9%) while ENA1 was present in only 10 out of the 106 total samples (9.4%) and ENA was not detected in any pasta sample. Regarding their distribution, ENB and ENB1 were present in 13 samples from Rabat, 11 from Kénitra, 10 from Casablanca, 10 from Salé, 8 from Fès, 8 from Agadir, and 7 from Témara. ENA1 was found in 6 samples from Rabat and 4 from Casablanca. In the positive samples, ENB concentrations ranged from 0.03 to 1.2 µg kg^−1^, ENB1 concentrations varied from 0.012 to 1 µg kg^−1^, and ENA1 was detected up to 0.03 µg kg^−1^. Recently, the presence of enniatins in pasta has been investigated. Indeed, two studies from Spain showed the occurrence of enniatins in different types of pasta. The first one reported the presence of enniatins in dry pasta where ENA was predominant [7]. In the second study, enniatins were found in all the analyzed samples of organic dry pasta, conventional dry pasta, and fresh pasta and ENB1 was predominant [27]. Even though enniatins were found to be frequent in the samples investigated herein and the percentage of positive samples was quite high (case of ENB and ENB1), the levels of enniatins found are lower compared to previous studies [27]. The extraction method used in our study showed a high sensitivity for *Fusarium* toxins specifically for ENA, ENA1, and ENB as compared to other studies in pasta. Indeed, the recoveries obtained were higher than 80%.

In the Mediterranean area, previous studies have shown the presence of enniatins in several cereal commodities, especially from Morocco. Indeed, the presence of *Fusarium* toxins was already reported in wheat grain with levels that ranged between 2.5 and 2570 µg kg^−1^ [22]. A previous study has shown also that breakfast cereals and infant cereals were contaminated with ENs, particularly ENA1 [28], while Sifou et al. reported the presence of ENs in rice samples with a contamination frequency that reached 50% of the total analyzed samples [29]. More recently, the presence of enniatins (ENB, ENB1, and ENA1) in couscous semolina from Morocco was also reported [17].

#### 2.2.6. Multi-Mycotoxin Occurrence in Pasta

From different toxicological studies, exposure to various mycotoxins together may lead to additive, and even synergistic toxic effects [30]. In the present survey, the multi-presence of several mycotoxins in the same pasta sample was observed. Indeed, among the positive samples, 73.6% (78/106) were found to be contaminated with at least one mycotoxin. A total of 39.6% of positive samples (42/106) were found contaminated simultaneously with 2 mycotoxins, while six mycotoxins co-occurred in only one pasta sample. In this work, the most frequent mycotoxins found in the pasta samples were enniatins (ENB and ENB1), ZEA, DON, T-2, and HT-2.

The multi-mycotoxin occurrence in cereals and derivatives has been surveyed in some Mediterranean countries. In wheat grain from Morocco, 51% of samples contained more than one mycotoxin [22]. While in Spain, it was reported that 65% of cereal derivatives samples showed a contamination by at least one mycotoxin [18]. Recently, a study performed in Italy showed that 80% of 27 pasta samples were contaminated with six to ten mycotoxins [31]. In Morocco, the co-occurrence of mycotoxins in couscous samples showed that ninety-six (96) out of ninety-eight (98) total samples (97.9%) were contaminated by at least one mycotoxin and that ENB and EB1 showed the highest incidence [17].

### 2.3. Risk Assessment

Risk assessment related to the dietary exposure of mycotoxins in pasta was calculated by estimating the PDIs for DON and derivatives (DON + 3-ADON + 15-ADON), ZEA, and T-2 + HT-2. However, as the Joint FAO/WHO Expert Committee on Food Additives has not established provisional maximum tolerable daily intakes (PMTDI) for Enniatins [32], PDIs were not estimated in this study for this kind of mycotoxins. It should be indicated that as there are no official data on pasta intake in Morocco, the PDIs estimated from pasta consumption in this investigation is only for orientation purposes. In 2004, according to the cereal professional association’s surveys, the consumption of pasta was estimated to be about 2 kg/person/year, however, since the consumption of pasta in the country is in escalation, the estimated value is 4.5 kg/person/year in 2016 [33].

The risk characterizations of these mycotoxins have been calculated using the previous equation based on the probable daily intakes of the Moroccan population, as shown in Table 4. The dietary exposure values through the consumption of pasta were estimated to be 0.073 µg kg^−1^ bw day^−1^ for the sum of (DON + 3-ADON + 15-ADON), 0.097 for the sum of (T-2 + HT-2), and 0.0002 for ZEA. Theses intakes values are equivalent to 0.08%, 7.3%, and 97% of the TDIs of ZEA, the sum of (DON + 3-ADON + 15-ADON), and the sum of (T-2 + HT-2) respectively. As shown, the highest PDI (97%) was obtained from the sum of (T-2 + HT-2). However, all estimated PDIs are below the TDIs set by FAO/WHO expert JECFA committees. Since the consumption of pasta is increasing in the country, so the presence of mycotoxins should be controlled because the exposure will get higher and might pose a health risk for the average consumer. High attention should be devoted to the exposure of regular consumers, such as children. For comparison, a study performed in Italy concerning the exposure assessment to DON through pasta consumption of different population groups in different scenarios concluded that no health concern was assessed for all consumers, the exposure being far below the TDI of 1 µg kg^−1^ bw day^−1^ [24].

## 3. Conclusions

In this paper, a multi-mycotoxin method analysis has been validated and applied to 106 pasta samples from Morocco to evaluate the presence of twenty mycotoxins. Most of the samples (93.4%) were contaminated with at least one mycotoxin. ENB and ENB1 showed the highest incidence followed by ZEA, DON, HT-2, and T-2. The multi-occurrence of mycotoxins in pasta samples was also found. Some samples exceeded the ML established by EU legislation, especially for DON. The risk of some of the mycotoxins studied was assessed and pasta consumption does not raise any toxicological concern in the general population because of the low mycotoxin exposure. However, some population groups (children and/or adolescent) could be exposed to a higher risk since they are considered more frequent consumers of pasta in the country.

## 4. Materials and Methods

### 4.1. Chemicals and Reagents

HPLC grade solvents (hexane, methanol (MeOH) and acetonitrile (AcN)) were purchased from Merck (Darmstadt, Germany). Deionized water (<18 MΩ cm resistivity) was obtained in the laboratory using a Milli-Q SP Reagent Water System (Millipore, Bedford, MA, USA).

Standards of mycotoxins including (aflatoxins (B1, B2, G1 and G2); enniatins (A, A1, B and B1); fumonisins (B1 and B2); nivalenol (NIV); deoxynivalenol (DON); 15-acetyldeoxynivalenol (15-ADON); 3-acetyldeoxynivalenol (3-ADON); zearalenone (ZEA); beauvericin (BEA); fusarenon X (FUS-X)) were purchased from Sigma Aldrich (Madrid, Spain). The T-2 and HT-2 toxins were provided from BiopureReferenzsubstanzenGmBH (Tulln, Austria). Finally, fumonisin B3 was provided by the Research Program “PROMEC” (Tygerberg) in South Africa. All stock solutions of mycotoxins standards were stored in glass-stoppered bottles in darkness at −20 °C until the final analysis. For the derivatization, the reagent composed of BSA (*N*,*O*-bis (trimethylsilyl) acetamide)/TMCS (trimethylchlorosilane)/TMSI (*N*-trimethylsilyimidazole) (3:2:3) was obtained from Supelco (Bellefonte, PA, USA).

### 4.2. Sample Collection

One hundred and six (106) pasta samples from different commercial brands were randomly purchased in retail shops and supermarkets from eight (8) cities in Morocco during the 2016 and 2017: Agadir (*n* = 11), Casablanca (*n* = 17), Fes (*n* = 11), Tanger (*n* = 10), Rabat (*n* = 20), Salé (*n* = 12), Kénitra (*n* = 15), and Témara (*n* = 10). The sample size of each sample was at least 2 kg. All samples were ground at <0.75 mm, divided into subsamples of 200 g, sealed in plastic bags, and stored at 4 °C until mycotoxin analysis.

### 4.3. Sample Preparation

The pasta sample was prepared and analyzed with an in-house validated method. Briefly, 5 g of pasta sample was weighed into a centrifuge tube of 50 mL. Ten (10) mL of water milliQ and 10 mL of AcN were added to the sample. The tube was vortexed for 5 min and shacked at 300 rpm for 30 min. Then, 1 g of NaCl and 4 g of MgSO4 were added and the tube was vortexed for 5 min. The tube was centrifuged at 4500 rpm for 10 min at 20 °C. The upper solution was evaporated to dryness under a nitrogen stream at 40 °C. Finally, the extract was dissolved with 1 mL of water/methanol (50/50, *v*/*v*). Before analysis, each sample was filtrated through a 0.22 µm PTFE filter and an aliquot of 500 µL was injected to the LC/MS/MS system, while the other aliquot was used for derivatization and GC/MS/MS analysis.

### 4.4. GC–QqQ-MS/MS Equipment

Prior to the GC/MS/MS analysis, the extracts were derivatized before their injection into the apparatus. For this, the extracts were evaporated to dryness under nitrogen, treated with 50 μL of BSA + TMCS + TMSI (3:2:3) and left for 30 min at room temperature. The sample was then diluted with hexane to obtain a volume of 250 μL and mixed with a vortex thoroughly for 30 s. Finally, the sample was washed with 1 mL of phosphate buffer (60 mM, pH 7) and the clear hexane layer was transferred into a vial for the analysis.

For mycotoxins determination, a GC apparatus system (Agilent 7890A, Agilent Technologies, Palo Alto, CA, USA) coupled with a triple quadruple mass spectrometer (Agilent 7000A) with an inert electron-impact ion source and an auto-sampler (Agilent 7693) was used. The mass spectrometer was operated in electron impact ionization (EI, 70 eV). Data were acquired and processed using the Agilent MassHunter version B.04.00 software (Palo Alto, CA, USA).

After derivatization, 1 µL of sample extract was injected at 250 °C in programmable temperature vaporization (PTV). Then, analytes were separated on a capillary column (HP-5MS 30 m × 0.25 mm × 0.25 μm) by using helium as the carrier gas with a fixed pressure value of 20.3 psi. The temperature was programmed (initially at 80 °C, and increased to 245 °C, at 60 °C min^−1^). The temperature was then increased to 260 °C after a 3 min hold time (at 3 °C min^−1^) and finally to 270 °C (at 10 °C min^−1^) and then held for 10 min. Analytes were eluted within 12 min, reaching the requirement for a high throughput determination.

For quantification purposes, the criteria set by the EC were achieved [34]. Indeed, for each analyte, two selected reaction monitoring (SRM) transitions for each compound were required. The most intense SRM transition was selected for quantification purposes. The specific MS/MS parameters for each mycotoxin were *m*/*z* 347/157–347/185 for HT-2, *m*/*z* 350/259–350/229 for T-2, *m*/*z* 289/73–379/73 for NIV, *m*/*z* 392/259–407/197 for DON, *m*/*z* 392/287–467/147 for 3-ADON, *m*/*z* 392/217–392/184 for 15-ADON, and *m*/*z* 450/260–450/245 for FUS-X.

### 4.5. LC/MS/MS Equipment

For LC/MS/MS analysis of mycotoxins, An LC triple quadruple mass spectrometer from Micromass (Manchester, UK) was used. The apparatus is equipped with an LC Alliance 2695 system (Waters, Milford, MA, USA), a quaternary pump, an autosampler, a pneumatically assisted electrospray probe, and a Z-spray interface. Additionally, the Mass Lynx NT software version 4.1 was used (Waters, Milford, MA, USA).

For the separation, a Gemini-NX C18 analytical column (150 × 2.0 mm I.D, 3.0 μm) supplied by Phenomenex (Barcelona, Spain) was used, the column was preceded by a cartridge C18. The mobile phase consisted of a mixture of solvents I (5 mM ammonium formate and 0.1% formic acid in methanol) and II (5 mM ammonium formate and 0.1% formic acid in water) at a flow rate of 0.25 mL/min. The elution gradient was established initially with 10% eluent B, increased to 80% in 1.5 min, then kept constant from 1.5 to 4 min, increased to 90% from 4 to 10 min, increased again to 100% from 10 to 14 min, and finally returned to the initial conditions and equilibrated during 10 min.

Analyses were performed in the positive ion mode. The main MS parameters were optimized and finally set as follows: nebulizer gas (GS1), 55 psi; auxiliary gas (GS2), 50 psi; curtain gas (CUR) 15 psi; capillary temperature 550 °C; ion spray voltage (IS) 5500 V. Nitrogen was used as the collision gas. The precursor-to-product ion transitions were *m*/*z* 313.3/241.3–284.9 for AFB1, *m*/*z* 315.3/259.0–288.4 for AFB2, *m*/*z* 329.7/243.3–311.1 for AFG1, *m*/*z* 331.1/313.0–245.0 for AFG2, *m*/*z* 722.4/334.3–352.3 for FB1, *m*/*z* 706.4/336.2–318.3 for FB2, *m*/*z* 706.4/336.2–318.3 for FB3, *m*/*z* 319.0/282.9–301.0 for ZEA *m*/*z* 657.3/196.1–214.0 for ENB, *m*/*z* 671.2/214.2–228.1 for ENB1, *m*/*z* 699.4/210.2–228.2 for ENA, *m*/*z* 685.4/214.2–210.2 for ENA1, and *m*/*z* 801.2/784.1–244.1 for BEA.

### 4.6. Method Validation

The method validation was carried out inclusive of the determination of accuracy, repeatability, sensitivity, and linearity. Recoveries were calculated at three different spiked levels 25, 50 and 100 µg kg^−1^ with three replicates (*n* = 3). The matrix effect (ME%) for each mycotoxin was determined according to the formula defined as the percentage of the matrix-matched calibration slope divided by the slope of the standard calibration and multiplied by 100. Matrix-matched calibration curves were used for the quantification in pasta. Accuracy was calculated through recovery studies, the spiked samples were left to stand for 2 h at ambient temperature before the extraction and three replicates were used for each spiking concentration. Precision was estimated by calculating the relative standard deviation (RSD) using the results obtained during the same day, and on three different days by the repeated analysis at the three spiked concentrations. Sensitivity was evaluated by the LOD and the LOQ, which were estimated for a signal-to-noise ratio (S/N) ≥3 and ≥10, respectively. Spiking was performed in 5-fold at two levels (10 and 100 µg kg^−1^). Together with the control samples, the fortified samples were processed using the extraction procedure described above. Aliquots of the control extracts were used to prepare the matrix-matched calibration using six concentration levels, between LOQ and 100 times LOQ. The same calibration standards were also prepared in water-methanol (50:50 *v*/*v*).

### 4.7. Mycotoxins Dietary Intake Calculation

For risk assessment of the studied mycotoxins, we have followed the deterministic approach. The exposure was estimated by the probable daily intake (PDI) which combines the average amount of mycotoxins found in pasta with the food consumption estimation in Moroccan adult populations. According to AMIPAC, the Moroccan consumption of pasta was approximately estimated at 2 kg per person per year [33]. The PDI (µg kg^−1^ per body weight (bw)/day) of each mycotoxin was calculated as indicated [18] in the following formula (A):

PDI = (M × C)/bw(A)

“M” is the mean level (µg kg^−1^) of each mycotoxin; “C” is the average consumption (g day^−1^) of pasta; and bw is the body weight used for the adult population (estimated at 70 kg). For the risk characterization of mycotoxins (% of relevant TDI), it was obtained by dividing the PDI previously calculated in the formula (A) with the tolerable daily intake (TDI) (µg kg^−1^ bw day^−1^) as indicated in the following formula (B):

%TDI = (PDI/TDI) × 100(B)

According to the safety guidelines of the FAO/WHO joint committees established for mycotoxins, the TDI values were 0.25 µg kg^−1^ bw day^−1^ for ZEA, 1.2 µg kg^−1^ bw day^−1^ for NIV, 0.1 µg kg^−1^ bw day^−1^ for the sum of T-2 to HT-2 and provisional maximum TDI (PMTDI) for fumonisins of 2 µg kg^−1^ bw day^−1^. The provisional maximum tolerable daily intakes (PMTDI) of DON + 3-ADON + 15-ADON was set at 1 µg kg^−1^ bw day^−1^ [35]. It should be indicated that the FAO/WHO joint expert groups have not specified the TDI for AFs since no safe limit can be set for confirmed carcinogenic chemicals. For this, FAO/WHO experts recommended that exposure through food should be reduced to *As Low As Reasonably Achievable* (ALARA). For Enniatins, BEA, and DAS, no PDI or PMTDI data are still fixed until now.

## Figures and Tables

**Table 1:** The sensitivity, recovery, linearity, and matrix effects using the liquid chromatography–tandem mass spectrometry (LC-MS/MS) analytical method.

Mycotoxin	LOD μg kg^−1^	Mean Recovery (%)			Intra-Day (RSD%)	Inter-Day (RSD%)	Matrix Effects SSE (%)	r^2^
		25 μg kg^−1^	50 μg kg^−1^	100 μg kg^−1^				
AFB1	0.01	108 ± 19	114 ± 8	78 ± 12	11	19	51	0.9914
AFB2	0.5	60 ± 17	71 ± 12	94 ± 11	8	17	45	0.9983
AFG1	0.03	59 ± 18	65 ± 11	93 ± 11	12	18	34	0.9935
AFG2	0.02	80 ± 12	63 ± 10	87 ± 12	6	12	64	0.9648
ENA	0.025	117 ± 16	92 ± 8	86 ± 9	10	16	69	0.9952
ENA1	0.025	112 ± 15	83 ± 11	86 ± 10	11	15	67	0.9890
ENB	0.03	96 ± 10	102 ± 9	116 ± 12	8	10	62	0.9910
ENB1	0.015	65 ± 11	64 ± 10	63 ± 18	6	16	27	0.9932
FB1	3	60 ± 10	67 ± 12	64 ± 17	6	12	55	0.9846
FB2	3	86 ± 13	47 ± 16	61 ± 17	8	19	58	0.9948
FB3	10	86 ± 15	67 ± 17	61 ± 15	11	13	58	0.9949
BEA	1	63 ± 14	60 ± 15	64 ± 17	6	12	66	0.9920
ZEA	0.5	103 ± 12	105 ± 11	98 ± 13	8	14	71	0.9860

LOD: limit of detection; RSD: relative standard deviation; SSE: signal suppression/enhancement; r^2^: regression coefficient.

**Table 2:** The linearity, recovery, and limit of detection and quantification (LOD/LOQ) using the gas chromatography–tandem mass spectrometry (GC-MS/MS) analytical method.

Mycotoxins	r^2^	LOD (μg kg^−1^)	LOQ (μg kg^−1^)	Recoveries (%) Spiking Levels (μg kg^−1^)			Intra-Day (RSD%)	Inter-Day (R SD%)
				25	50	100		
DON	0.9993	0.5	1	85 ± 16	89 ± 11	86 ± 8	7	11
3-ADON	0.9998	1	3	95 ± 11	72 ± 11	62 ± 12	9	11
15-ADON	0.9966	1	3	69 ± 12	69 ± 15	62 ± 21	13	15
NIV	0.9972	1	2.5	97 ± 4	86 ± 14	65 ± 9	2	14
T-2	0.9993	2	5	80 ± 11	69 ± 16	62 ± 14	5	20
HT-2	0.9885	2.5	5	69 ± 10	68 ± 9	81 ± 11	15	19
FUS X	0.9429	5	10	84 ± 9	73 ± 12	67 ± 20	10	12

r^2^: regression coefficient; RSD: relative standard deviation.

**Table 3:** The multi-mycotoxin occurrence in the analyzed pasta samples.

Mycotoxin	Agadir (*n* = 11)		Casablanca (*n* = 17)		Fes (*n* = 11)		Tanger (*n* = 10)		Rabat (*n* = 20)		Sale (*n* = 12)		Kenitra (*n* = 15)		Temara (*n* = 10)	
	Positive Samples	Range (μg kg^−1^)	Positive Samples	Range (μg kg^−1^)	Positive Samples	Range (μg kg^−1^)	Positive Samples	Range (μg kg^−1^)	Positive Samples	Range (μg kg^−1^)	Positive Samples	Range (μg kg^−1^)	Positive Samples	Range (μg kg^−1^)	Positive Samples	Range (μg kg^−1^)
AFB1	1	0.25	0	n.d	0	n.d	0	n.d	1	0.01	0	n.d	0	n.d	0	n.d
ENA1	0	n.d	4	0.025–0.03	0	n.d	0	n.d	6	0.025–0.03	0	n.d	0	n.d	0	n.d
ENB	8	0.05–0.09	10	0.03–1.2	8	0.05–0.3	5	0.04–1	13	0.09–1	10	0.05–1	11	0.03–0.09	7	0.03–0.07
ENB1	8	0.06–0.012	10	0.7–1	8	0.2–0.6	5	0.02–0.6	13	0.3–1	10	0.4–0.9	11	0.2–0.7	7	0.2–0.8
ZEA	9	0.6–1	13	0.9–3	3	0.5–0.9	8	2	12	1–2.4	5	0.5–2	2	0.5–0.9	3	0.7–1
DON	2	292–900	10	271–830	2	479–668	4	719–770	9	16–538	8	137–671	3	23–138	5	232–301
3-ADON	1	3.03	0	n.d	0	n.d	0	n.d	0	n.d	0	n.d	0	n.d	0	n.d
HT-2	2	7–31	8	9–419	3	13–19	6	4–22	7	9–75	4	38–88	4	3–9	3	4–27
T-2	2	4–21	3	11–33	2	13–19	2	4–50	2	8–48	2	9–47	2	12–22	2	5–33

*n*: number; n.d: Not detected.

**Table 4:** The estimated exposure of the studied mycotoxins.

Mycotoxin	Cm (µg kg^−1^ pasta) *	K (g person^−1^ day^−1^) **	PDIm (µg kg^−1^ bw day^−1^)***	TDI (µg kg^−1^ bw day^−1^)	% TDI
DON + 3-ADON + 15-ADON	440.95	11.6	0.073	1	7.3
HT-2 + T2	58.65	11.6	0.097	0.1	97
ZEA	1.20	11.6	0.0002	0.25	0.08

* Cm: Mean content of a mycotoxin (µg kg^−1^ pasta). ** K: Average consumption of the commodity (g person^−1^ day^−1^). *** PDIm: Probable daily intake (µg kg^−1^ bw day^−1^) for each mycotoxin. TDI: Tolerable daily intake.
